# Microbial Inoculants in Sustainable Agriculture: Advancements, Challenges, and Future Directions

**DOI:** 10.3390/plants14020191

**Published:** 2025-01-11

**Authors:** Alondra María Díaz-Rodríguez, Fannie Isela Parra Cota, Luis Alberto Cira Chávez, Luis Fernando García Ortega, María Isabel Estrada Alvarado, Gustavo Santoyo, Sergio de los Santos-Villalobos

**Affiliations:** 1 Laboratory of Microbial Resource Biotechnology, Department of Agronomic and Veterinary Sciences, Sonora Institute of Technology, 5 de Febrero 818, Centro, Ciudad Obregón 85000, Sonora, Mexico; alondramdr07@gmail.com (A.M.D.-R.); luis.cira@itson.edu.mx (L.A.C.C.); mestrada@itson.edu.mx (M.I.E.A.); 2 Norman E. Borlaug-INIFAP Experimental Field, Norman E. Borlaug Km. 12, Ciudad Obregón 85000, Sonora, Mexico; fipc04@gmail.com; 3 Laboratory of Learning and Research in Biological Computation, Department of Genetic Engineering, CINVESTAV-IPN, Km 9.6 Libramiento Norte, Irapuato 36824, Guanajuato, Mexico; luisfernandgar@gmail.com; 4 Institute of Chemical Biological Research, Michoacan University of San Nicolas de Hidalgo, Morelia 58030, Michoacan, Mexico; gustavo.santoyo@umich.mx

**Keywords:** plant growth promotion, bioformulation, sustainability, microbial consortia, multi-omics

## Abstract

The rapid growth of the human population has significantly increased the demand for food, leading to the intensification of agricultural practices that negatively impact the environment. Climate change poses a significant threat to global food production, as it can disrupt crop yields and modify the lifecycle stages of phytopathogens and pests. To address these challenges, the use of microbial inoculants, which are bioproducts containing beneficial microorganisms known as plant growth promotion microorganisms (PGPMs), has emerged as an innovative approach in sustainable agriculture. This review covers the isolation and identification of beneficial strains, the screening and selection process, the optimization of production techniques, and the importance of quality control and field testing. It also discusses the key points for the development and formulation of high-quality microbial inoculants, as well as highlights their advancements, current challenges, and future directions for research and application.

## 1. Introduction

The rapidly growing human population has significantly increased the demand for food in the last decade. However, intensive non-sustainable agricultural practices place food security, the economy, and the environment at risk worldwide. Climate change poses a significant threat to global food production, as rising temperatures, changing precipitation patterns, and more frequent extreme weather events can modify the lifecycle stages and the development rates of phytopathogens and pests [1] and disrupt crop yields [2]. According to several studies, under the most severe climate change scenario and without adaptation strategies, simulated crop yield losses range from 7 to 30% during the mid-century (2040–2069) [2,3,4].

Thus, farmers have relied on the use of high amounts of synthetic agro-inputs to fertilize crops and maintain phytosanitary control, which negatively impacts human and environmental health. According to the FAO (2021), synthetic nitrogen fertilizers are responsible for approximately 38% of agricultural emissions derived from the release of soil nitrous oxide (N_2_O). The impacts extend to the economic and social spheres since the agriculture-derived nitrogen emissions account for economic losses of around USD 200 billion annually, and the costs for human health and the impact on aquatic and terrestrial ecosystems amount from USD 400 billion to USD 4000 billion annually.

In this manner, an innovative approach to sustainable agriculture is the use of microbial inoculants. These bioproducts, containing plant growth promotion microorganisms (PGPMs), contribute to improving soil health, enhancing nutrient cycling, and boosting plant resilience against environmental stressors, pests, and diseases. By fostering beneficial microbial communities in the soil, microbial inoculants increase nutrient availability, improve soil structure, and reduce the dependency on chemical fertilizers and pesticides, which have detrimental effects on the environment [5,6]. The use of these bioproducts aligns with the principles of sustainable agriculture by reducing environmental pollution, lowering greenhouse gas emissions, and improving soil biodiversity, ultimately contributing to more resilient and productive farming systems in the face of climate change.

The use of microbial inoculants in agriculture has a long history, dating back to the late 19th century when researchers began exploring the potential of nitrogen-fixing bacteria to improve crop yields. In 1896, the USA introduced the first commercially produced microbial inoculant, “Nitragin,” which initially used gelatin and later a nutrient medium as carriers for bacterial cells. Due to high mortality rates, these carriers were eventually replaced by peat, which remained the preferred carrier until the late 1990s [7]. In the 1950s, the use of rhizobial inoculants expanded to developing countries, though adoption was limited due to factors like poor quality control and lack of access [8].

Then, between the 1980s and 2000s, the production and application of bacterial inoculants evolved, with the diversification of inoculant strains and advancements in formulations. In Brazil, the first liquid inoculant was approved by the Ministry of Agriculture for commercial use in 2000, and a decade later, nearly 80% of the inoculants sold in the country were in liquid formulations, a trend also seen in Argentina [7]. In 2008, the first biofungicide was developed in Mexico, which is currently registered for the control of anthracnose in papaya, avocado, and citrus, and is available in both liquid and wettable powder formulations [9].

The integration of omics sciences in the field of agriculture for microbial inoculants has been an ongoing process, with significant advancements in the last two decades, enabling the development of more efficient strains and a more comprehensive characterization of plant–microbe interactions and the complex mechanisms involved [10], leading to the optimization of inoculant formulations and application strategies for improved crop productivity and sustainability [11].

As the demand for sustainable agricultural solutions continues to grow, the production and use of bacterial inoculants are expected to expand. However, the widespread adoption and effective use of microbial inoculants face several current and future challenges. These include ensuring consistent product quality, optimizing application methods, adapting inoculants to local environmental conditions, and overcoming economic, regulatory, and knowledge barriers. Addressing these challenges through continued research, innovation, and targeted policies will be crucial for unlocking the full potential of microbial inoculants in supporting sustainable agriculture and enhancing global food security. This review aims to analyze key points for the development and formulation of high-quality bacterial inoculants, as well as highlight their advancements, current challenges, and future directions for research and application in the field of bacterial inoculant production.

## 2. Microbial Inoculant Production

### 2.1. Isolation and Identification of Beneficial Strains

Selecting microorganisms for bioformulation development is a crucial initial step in the bioformulation startup process. To select potential candidates, various microbial sources such as soil, water, or plant tissues are used for isolation [12]. For example, to find potential antagonists for the control of *Fusarium* head blight in wheat, isolations have mostly been conducted from wheat ears, as the most vulnerable stage to the disease is during anthesis [13].

Recent studies have reported the benefits of using native microorganisms as inoculants to enhance plant growth, suggesting that these strains are already adapted to the local agro-climatic conditions [14,15,16]. For example, desiccation-tolerant strains of white clover-nodulating strains of *Rhizobium leguminosarum* bv. *trifolii* was successfully isolated from sites where soil moisture deficits were common [17]. This increases the chances of inoculum survival and potentially enhances plant development, even under previously encountered stresses [18,19]. Li et al. (2023) evaluated the impact of an inoculant based on *Bacillus paralicheniformis* and *B. subtilis*, isolated from wheat, on the growth and stress responses of three wheat varieties under drought conditions, as well as its effect on soil microbial communities [20]. The study reported increased plant shoot weight, enhanced nitrogen enzyme activity, and the establishment of the inoculant as keystone taxa in the soil, leading to a higher relative abundance of other *Bacillus* strains. These changes contributed to improved wheat performance under drought conditions.

**Figure 1:**
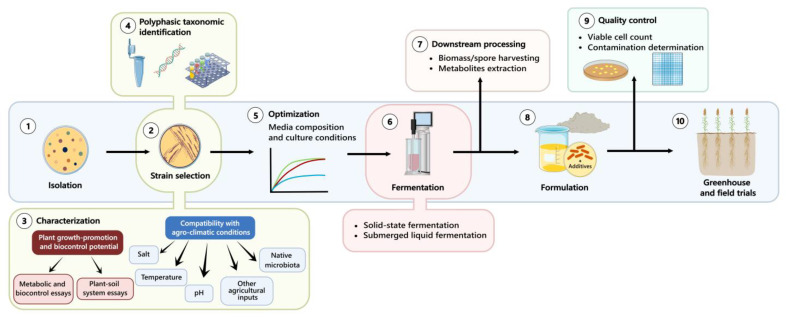
Schematic representation of the production process for microbial inoculants. The process begins with the isolation of microbial strains from environmental samples (1), followed by strain selection (2) based on plant-growth-promotion and biocontrol potential (3), and the compatibility with agro-climatic conditions (3). Selected strains undergo polyphasic taxonomic identification to confirm their identity (4). Subsequently, optimization of media composition and culture conditions is performed to enhance growth and productivity (5). The microbial strains are then mass-cultured through fermentation, employing either submerged liquid or solid-state fermentation techniques (6). Downstream processing involves harvesting biomass, spores, or secondary metabolites as needed (7). The microbial product is stabilized through formulation by adding carriers or additives to maintain viability and extend shelf life (8). The quality control stage ensures product consistency, with viable cell counts and contamination checks (9). Finally, the inoculant undergoes greenhouse and field trials to validate its effectiveness under controlled and real-world agricultural conditions (10).

In the next step, for candidate selection, these are preliminarily evaluated using a rapid-throughput approach with standardized, simple, and cost-effective experiments to determine their ecological fitness [Figure 1 (2–3)]. One main filter can be the biosafety of the strains for humans, animals, and the environment. At present, however, there are no internationally harmonized, reliable protocols to evaluate the safety of these bacterial strains. Some common methods used are the discard of strains that grow at the human body temperature (36 °C), biochemical essays, antibiotic sensitivity methods, virulence testing in animal models, and high-throughput sequencing to identify pathogenic genes [21].

Preliminary in vitro experiments are performed that can pre-select promising candidates with specific growth promotion characteristics and modes of action against pathogens. This preliminary step may include, nitrogen fixation, phosphate solubilization, siderophore production, hydrolytic enzymes, competition for nutrients and space, phytohormone synthesis, and evaluation of stress tolerance, among others [22,23]. In addition, the compatibility of the candidates with agrochemical products such as fungicides, insecticides, and fertilizers is also performed at this step if their potential use in certain integrated cropping systems is envisaged [24,25,26]. For example, a study by Mishra & Sundari (2015) found that a consortium of PGPMs could provide better growth promotion in sorghum even in the presence of the organophosphate pesticides malathion and methyl parathion [14]. However, demonstrating the potential mechanism under artificial conditions, typically on nutrient-rich media, does not ensure that these mechanisms will function similarly in crop environments, such as nutrient-limited soils [27]. This rapid screening step helps to narrow down the number of candidates that will be subjected to more labor-intensive and resource-demanding bioassays later. Therefore, the efficacy of candidates is assessed in bioassays with plants or plant tissues, mimicking the environmental conditions in which the microbial inoculant will be applied [28].

The next step involves identifying isolates that fulfill the basic selection criteria regarding ecological fitness at the species level using DNA sequence analysis [Figure 1 (2–4)]. A polyphasic taxonomic approach including genotypic, phenotypic (physiological, morphological, and biochemical), and chemotaxonomic data are strongly recommended to accurately affiliate microorganisms [29]. In addition, the integration of genome mining in this approach can provide access to relevant data on microbial traits and metabolic capabilities, including their ability to produce bioactive compounds [30,31]. This information can provide valuable insights into the ecological fitness and functional potential of the candidate inoculant strains.

### 2.2. Culture Media, Conditions Optimization, and Mass Cultivation Methods

Microbial inoculants are produced through the fermentation process, which involves the cultivation of microorganisms in a specific nutrient medium to produce specific metabolites, enzymes, or biomass [32,33]. Depending on the type of organism, production is evaluated in solid-state fermentation (SSF), commonly used for filamentous fungi, or submerged liquid fermentation, commonly used for bacteria and yeasts [34]. However, before fermentation, the optimization of culture medium and fermentation conditions is crucial to obtain high yields of the product of interest (i.e., biomass, spores, metabolites, enzymes, among others) as they impact not only the later growth promotion and biocontrol effects in the crops, but also the production cost, capital utilization efficiency, and account for any losses in subsequent downstream processes such as cell harvesting and bioproduct formulation [35] [Figure 1 (5–7)].

To maximize the production process, it is essential to determine the optimal media and culture conditions for each potential strain. Various techniques can be employed, ranging from classical “one-factor-at-a-time” to modern statistical and mathematical methods, to identify limiting nutrients in the media and optimal condition parameters such as dissolved oxygen, temperature, pH, and mixing speed [36]. Some studies suggest that combining different optimization techniques yields the best results. For instance, Shi et al. (2024) reported that using a Plackett–Burman design and the response surface methodology increased the viable count of *B. velezensis* BHZ-29 from 7.83 × 10^9^ to 3.39 × 10^10^ CFU/mL and the antibacterial titer in biocontrol essays from 111.67 to 158.85 mm/mL when the optimal media and conditions were applied in the fermentation process [33].

Key factors include the sources of carbon and nitrogen, along with their cost and availability (Table 1). Microorganisms can easily assimilate simple sugars like glucose, sucrose, and lactose, but, in some cases, manipulating growth-limiting medium components, organic acids, or metabolite production can be managed [36]. Additionally, media derived from agro-industrial wastes, which are economically attractive, have been successfully tested for industrial inoculant production [34,37]. For example, Ahsan et al. (2022) evaluated the fermentation filtrate of *B. amyloliquefaciens* BAM strain produced with different carbon sources in the biocontrol against *Cercospora arachidicola*, reporting a decrease in inhibition rate when lactose and sucrose were used, and the greatest inhibition rate (81%) when semolina flour was used [38]. On the other hand, as described by Monteiro et al. (2014), using a culture medium with chemically defined ingredients is of great importance to obtain reproducible and homogeneous cultures [39]. This ensures consistency in the composition of the medium, allowing for reliable and standardized results in the fermentation process.

After the selection of a suitable strain as well as medium and optimal growth conditions at the laboratory level, the next step is scale-up, which is typically carried out in two stages: pilot-scale production and large-scale production; this involves using bioreactors of different sizes [Figure 1 (6)]. The stirred-tank bioreactors for liquid fermentation have been successful in bacterial strain production due to their efficient mixing, scalability, versatility, process control, and ease of sterilization [45].

The fermentation can be carried out in batch, fed-batch, or continuous modes to optimize productivity and meet specific process requirements. Batch fermentation is a discontinuous process in which nutrients are supplied to the microorganisms only once at the beginning of the fermentation. Once the nutrients are depleted and the product of interest is produced, the entire content of the fermenter tank is harvested for the next step of the processing [46]. However, in fed-batch fermentation processes, the rate of nutrient addition of a limiting nutrient is controlled to regulate the reaction rate to reach a sufficiently high biomass or high concentration of phyto-stimulating metabolites [47,48]. Monteiro et al. (2014) achieved significant results using a 2 L stirred-tank bioreactor with *Bacillus* strains, reaching 6.3 × 10^9^ spores/mL in batch culture, and 3.6 × 10^10^ spores/mL in fed-batch culture [39]. In another study, Petersen et al. (2021) reported that the optimized fed-batch process achieved a maximum cell dry weight of 8.5 g/L of *Paraburkholderia* sp. SOS3, which is among the highest reported for plant growth-promoting bacteria in stirred-tank bioreactors [48].

Lastly, in continuous fermentation, the bioreactor is operated with a constant inflow of fresh medium and outflow of fermented broth; therefore, cells are maintained in an exponential growth phase due to the replenishment of consumed nutrients and removal of toxic metabolites. This type of fermentation is often used for the production of primary metabolites or biomass, where a steady supply of the product is desired [47]. In the case of SSF, the fermentation is performed in various containers, such as plates, beakers, flasks, bottles, bags, trays, and column bioreactors, allowing for the solid substrate to be inoculated with the desired microorganisms and maintained under the optimal conditions for their growth and product formation [49,50]. However, at the industrial scale, there are some challenges, mainly due to heat and mass transfer limitations and the lack of well-established scale-up criteria [34,49]. Regardless of the fermentation mode, the downstream processing and formulation of the microbial inoculants are also crucial steps that impact the product quality, shelf life, and commercial viability.

### 2.3. Formulation and Stabilization (Liquid Formulations and Solid Formulations)

A microbial inoculant is composed of one or more PGPM as the active ingredient and a carrier material together with other substances called additives that have the potential to increase the survival and efficacy of the microorganism [51]. Microbial inoculants are formulated using various liquid and solid carriers to improve their stability and shelf life [Figure 1 (8)].

Bioformulation based on solid carriers can produce suitable inorganic/organic or synthetic carriers viz. peat, charcoal, clays, talc, wheat bran, vermiculite, perlite, silicates, etc. [34] and these can be formulated in powders or granules [52]. A simple technique used for the preparation is the mix of the microorganisms with the solid support and air-drying them overnight to retain 15–20% moisture content [12], which is essential to lower microbial metabolic activities. However, using complex organic matter can present great chemical variability and, consequently, it is difficult to maintain the same quality in all batches [53]. Another common technique for solid formulation is spraying or lyophilization. These techniques enable high microbial survival rates and storage for long periods at room temperature. However, drying is not well tolerated by many PGPMs, especially for non-spore-forming bacteria, which might tend to lose viability after dehydration and storage [54,55].

Liquid formulations use culture broths or formulations based mainly on water, mineral, or organic oils. Compared to traditional solid carrier-based formulations, liquid formulations allow for better sterilization control and can maintain higher viable cell counts of beneficial microbes throughout the culture cycle, allowing for lower application rates [53]. In addition, it can extend its shelf life up to 19–25 months, with suitable additives such as polymers (PVP, polyethylene glycol, sodium alginate, glycerol, Arabic gum, trehalose), adjuvants (CMC, xanthan gum, carrageenan), and surfactants (polysorbate 80, 40 and 20) [56,57].

The choice of formulation depends on the specific microorganism, its intended application, and the desired characteristics of the final product. The resulting formulated inoculum produced is then stored under commercially acceptable conditions and also under stress conditions, and viability and efficiency are tested at regular intervals [57]. One recommendation is to run both types of experiments in parallel to reduce costs. The shelf life of the candidates after the whole process is used as a main criterion of bioformulation selection.

### 2.4. Quality Control and Field Testing

Rigorous quality control measures during the manufacturing process are critical to ensure consistency and reliability in microbial formulations [Figure 1 (9)] [12]. To ensure quality, standards should be established for parameters like viable cell counts, purity, and effectiveness, and this should be implemented throughout the production process, from the initial culture selection to the final formulation and packaging [58].

Another challenge is the variability in the performance of microbial inoculants under field conditions [59]. Unlike broad-spectrum chemical inputs, microbial inoculants can be selective and targeted, which can lead to inconsistent results. Robust field evaluation and careful monitoring measures are needed to ensure that the bioformulated and stored product performs over several seasons under a range of climatic/geographical locations and soil types [Figure 1 (10)] [52]. Besides the effects on the plants, additional assessments should be performed about the persistence of the applied bioinoculants and their effect on non-target organisms in the field. A strain-specific qPCR is a powerful tool for the required long-term studies on population dynamics and tracking bioinoculants in the field [60].

The evaluation of the efficacy of bioformulations is also essential for estimating their economic use; therefore, different conditions are tested: application times, intervals, and concentrations [61]. Field-scale variability requires multi-year, multi-location field data to achieve sufficient statistical significance for the separation of treatment effects and ensure robust analysis; to detect a 10% difference in crop yield may require between 9 and 28 replicates [62]. Additionally, establishing untreated control groups and reference products in trials can provide a benchmark for evaluating the performance of new bioformulations. Some countries have their standards and recommendations for performing efficacy evaluations [63]; however, there is a gap in developing countries, especially in Latin America where these bioproducts remain largely regulated by chemical fertilizer and pesticide laws [64]. For example, the European and Mediterranean Plant Protection Organization has set up several guidelines (http://pp1.eppo.int/ accessed on 9 January 2025) for the evaluation of direct efficacy, number of trials, and phytotoxicity assessments for biocontrol agents products [65]. Finally, after field validation, the bioformulation needs registration through a patent before commercialization.

## 3. Advancements and Future Directions

### 3.1. Biotechnological Approaches for Enhanced Microbial Inoculant Efficiency

The development of precise genetic engineering tools and the availability of complete genome sequences has facilitated the development of new microbial formulations. Techniques used in genetically modified microorganisms include cloning target genes or gene clusters into bacterial chassis, gene transfer, in vitro mutagenesis, and genetic recombination [66]. This allows the bacteria to express heterologous proteins or molecular compounds that can benefit plants, such as by stimulating plant growth or inhibiting the growth of harmful microbes [67]. Wu et al. (2018) developed a gene repressor system based on xylose-induced CRISPR interference in *B. subtilis*, aimed to downregulate the expression of the zwf, pfkA, and glmM genes that control the major competing reactions of N-acetylglucosamine (GlcNAc) synthesis. They achieved a five-fold increase in GlcNAc yield, reaching 103.1 g/L, with a titer of 1.17 g/L/h in a 3 L fed-batch bioreactor [68].

Genes responsible for unwanted characteristics like toxin production or excessive biofilm formation can be knocked out using CRISPR/Cas9 to improve the safety and efficacy of microbial inoculants. However, the practical implementation of this approach still faces challenges, and the safety and regulatory aspects require rigorous evaluation. Gene knockout also allows the linkage of resulting phenotypes with gene functions [69]. In the past, genome engineering tools were primarily limited to model organisms; however, with the wide range of available options today, it is possible to knock out genes in non-model strains, including PGPM. This strategy has been useful in confirming the expression of plant growth-promoting traits in situ. For instance, *B. amyloliquefaciens* SQR9 is a well-known plant growth-promoting bacteria of cucumber. The deletion of the yhcX gene in this strain (SQR9DyhcX), which is an essential gene for auxin biosynthesis, reduced its ability to promote cucumber growth, provides evidence that SQR9 promotes cucumber growth by auxin production [70].

Combining multiple PGPMs into consortia-based inoculants could also allow the combination of different potential mechanisms without the need to resort to genetic engineering, enhancing their performance and adaptability in the field compared to single-strain inoculants [71], compensating for traits lacking in others, and leading to enhanced overall effects. For instance, the application of a consortium containing strains of *Erwinia* sp. EU-B2SNL1 (N-fixer), *Chryseobacterium arthrosphaerae* EU-LWNA-37 (P-solubilizer), and *Pseudomonas gessardii* EU-MRK-19 (K-solubilizer) enhanced the growth and physiological parameters including root/shoot length and biomass, chlorophyll, carotenoids, phenolics, flavonoids and soluble sugar content on barley crop [72]. Other combinations could be plant-growth-promoting bacteria (PGPB) plus arbuscular mycorrhizal fungi (AMF); Pacheco et al. (2021) reported the inoculation of *Rhizoglomus irregulare* and *P. putida* produced around 50% more plant biomass per unit of P taken up, enhancing plant internal P use efficiency under field conditions, while being compatible with conventional agricultural practices [73]. In addition, microbial consortia can facilitate the establishment and functioning of target beneficial strains through synergistic interactions; for example, the synergistic interaction between *B. velezensis* SQR9 and *P. stutzeri* is mediated by metabolic interactions and biofilm formation, and it has been reported that their inoculation can promote plant growth and help alleviate salt stress in cucumber plants [74]. In another study, liquid chromatography-mass spectrometry/mass spectrometry (LC-MS/MS) has shown that co-culture of *B. amyloliquefaciens* ACCC11060 and *T. asperellum* GDFS1009 increases the production of specific biocontrol substances and amino acids compared to their pure cultures [75]. Despite the advantages mentioned, the application of microbial consortia comes with some challenges, with similar disadvantages as single species approaches, such as the development of optimal formulation, ensuring the viability and compatibility between strains during production, storage, and application, and the complexity of interactions that can be influenced by biotic and abiotic factors [76].

### 3.2. Novel Technologies in Bioprospection and Evaluation Effectiveness

**Figure 2:**
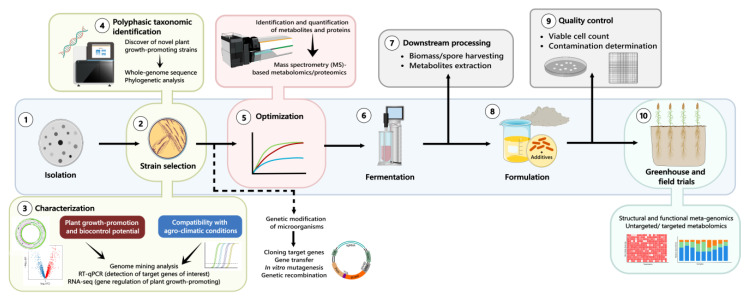
New omics and biotechnological approaches in microbial inoculant production. This figure highlights the integration of omics and biotechnological tools throughout the microbial inoculant development process to enhance efficiency and effectiveness. Advanced techniques such as whole-genome sequencing, metabolomics, proteomics, and transcriptomics are utilized for strain identification, optimization, and evaluation of plant growth-promoting potential. These approaches ensure precise strain characterization, improved metabolite/biomass production, and compatibility with agro-climatic conditions. Combined with genetic modifications, fermentation, and quality control processes, these innovations enable the development of robust inoculants for sustainable agricultural applications.

In general, culture-dependent methods in bioprospection studies are useful for identifying potential modes of action underlying plant growth promotion. However, it is important to note that the in vitro conditions used to assess plant growth-promoting traits may differ from those found in situ [77]. Cutting-edge developments in high-throughput sequencing and multi-omics technologies, including genomics, transcriptomics, metabolomics, and proteomics, have played a crucial role in detecting the activity of plant growth promoting and biocontrol traits at different functional levels, such as genes, transcripts, proteins, and metabolites (Figure 2) [78].

For instance, the use of next- and third-generation sequencing technologies and genomic analysis has enabled the discovery of novel plant growth-promoting strains [79]. A study combined culture-dependent, phylogenetic analysis and whole-genome sequencing [80] to isolate, characterize, and identify a *B. cabrialesii* TE3^T^, as a new bacterial species, an endophytic bacterial strain, which exhibited potent antifungal activity against various plant pathogens under in vitro assays, as well as genes involved in the biosynthesis of antimicrobial compounds and genes linked to plant growth promotion through mechanisms like phytohormone production and nutrient solubilization.

Currently, computational resources such as KEGG, SEED, RefSeq, Enzyme Commission, Bio/MetaCyc, Pfam, GO, InterPro, and RAST enable the functional annotation of genomes and classification of genes involved in various biological processes [81]. However, these resources have been outperformed by the emergence of tools, such as anti-SMASH [82], BiG-SCAPE [83], and PLaBAse [84], which allows the mining of genes involved in the biosynthesis of antimicrobial metabolites and plant growth promotion mechanisms from genomes or metagenome-assembled genomes (MAGs).

Early transcriptomic studies were performed with the aim of rapid detection and quantification of target microbial species or genes of interest using reverse transcription quantitative real-time PCR (RT-qPCR). With this approach, gene selections to monitor their expression are commonly made based on prior observations of in vitro plant growth-promoting traits conducted during the screening in the bioprospection stage and genome mining analysis [78]. This strategy has been useful in monitoring the expression of genes involved in plant growth-promoting traits such as nitrogen fixation (nifH) [85], phosphorus solubilization (PhoR, PhoA) [86], and indoleacetic acid production (ipdC, aspC); antimicrobial metabolites production involved in phytopathogen inhibition such as Phenazine (phzF) and Pyrrolnitrin (prnD) [87], stimulation of the induced systemic resistance (ISR) through pathogenesis-related gene (PR-1, 2), plant defensive chitinase (Chit-1), and β-1, 3-glucanase (Glu-2) genes [88]. The PR-1 and PR-2 genes are key players in plant defense mechanisms, encoding proteins that are upregulated during the defense response against various pathogens. PR-1 is a small protein associated with systemic acquired resistance (SAR) and is involved in enhancing the plant’s ability to resist pathogen invasion, particularly by acting as a marker for pathogen-induced immune responses. PR-2 encodes for a β-glucanase enzyme that hydrolyzes glucan chains in the cell walls of pathogens, aiding in their degradation and enhancing the plant’s defense. Additionally, chitinases like Chit-1, which degrade chitin, a key component of fungal cell walls, play a vital role in protecting plants against fungal infections, while glucanases such as Glu-2 provide a broader defense mechanism against both fungal and bacterial pathogens by breaking down β-glucan polymers. Together, these enzymes and PR genes contribute significantly to a plant’s ability to resist infections and promote resilience, making them essential for the application of biocontrol agents in sustainable agriculture [89]. Even though this approach provides evidence of plant growth promotion activities in situ, it lacks the resolution required to comprehensively analyze the transcriptomic landscape and, therefore, explore novel mechanisms that cannot be discovered following this approach [78].

In this manner, RNA sequencing methods have become the standard method to explore transcriptomic responses [90] of the interaction of PGPMs and plants, providing valuable insights into the complex gene regulations that promote plant growth since it provides an unbiased, genome-wide view of gene expression. For example, transcriptomic analysis has revealed that inoculation with salt-tolerant bacteria significantly influences wheat gene expression involved in various metabolic pathways and stress response mechanisms, with effects more prominent in leaves than roots [91]. Transcriptome analysis of chrysanthemum has also demonstrated enhanced growth and quality with co-inoculation of multiple PGPR strains compared to single strain inoculation, since the co-inoculation induced gene expression in cellular metabolism, signal transduction, and beneficial substance synthesis in the plant [92]. Transcriptomic studies have revealed that PGPMs enhance the expression of genes associated with salicylic acid (SA)-mediated systemic acquired resistance (SAR), including pathogenesis-related (PR) genes such as PR-1. In addition to their role in fungal inhibition, these genes are instrumental in inhibiting viral replication and spread within the plant. Additionally, PGPMs stimulate the jasmonic acid (JA) and ethylene (ET) signaling pathways, which synergistically enhance antiviral defenses. For example, studies have demonstrated upregulation of the ACS2 gene, which is involved in ethylene biosynthesis, and other defense-related transcripts like LOX2 and OPR3 linked to jasmonic acid signaling, contributing to enhanced plant resilience. In the absence of chemical antiviral agents, leveraging PGPMs offers a sustainable and environmentally friendly strategy for managing viral infections in crops while simultaneously promoting plant health and growth [93].

Metabolomics-based screening assay analysis can be used to detect beneficial microbial strains. The analysis can uncover novel bioactive compounds produced by microbial inoculants, such as antimicrobials, phytohormones, and nutrient-mobilizing agents [11,94,95]. This allows efficient prioritization of strains with desirable metabolite profiles. Untargeted metabolomics can provide an overview of the metabolic changes in the rhizosphere in response to microbial inoculation [96]. By comparing the metabolomes of inoculated and non-inoculated plants, researchers can identify differentially abundant metabolites that may be involved in plant growth and health [97].

**Figure 3:**
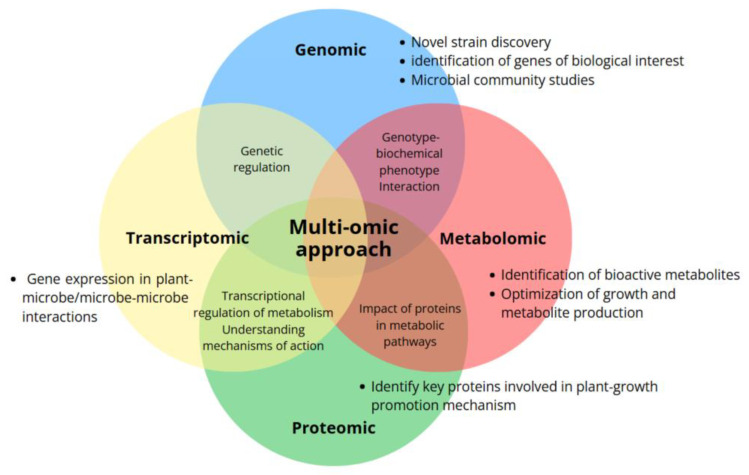
Application of multi-omics approach in the study and development of microbial inoculants.

Multi-omics approaches enable the study of the complex interactions between bioinoculants, plants, and pathogens [98]. By analyzing the genomic, transcriptomic, and metabolomic profiles of diverse microbial isolates researchers can identify those with the most desirable plant growth-promoting traits, or even the most effective combinations for specific crops and environmental conditions. This holistic understanding of plant–microbe interactions can be used to optimize inoculant formulations and application strategies (Figure 3). Villa-Rodriguez et al. (2021) explored the inhibition of the fungal pathogen *Bipolaris sorokiniana* by *B. cabrialesii* TE3^T^ using a genomic-metabolomic approach. Genome mining revealed the biosynthetic potential of strain TE3^T^ to produce a wide spectrum of antifungal and antibacterial metabolites, and bioactivity-guided LC-ESI-MS/MS analysis determined that a lipopeptide complex of surfactin and fengycin homologs was responsible for the antifungal activity [11].

Besides unraveling the mechanisms of action of microbial inoculants, the study of the impact of bioinoculants on the soil microbial communities is a crucial aspect for evaluating their efficiency in plant growth and field yield. However, the effects of bioinoculants on resident microbial communities are complex and can be mediated by inoculant type (bacterial or fungal), diversity (single strain vs. mixed), and disturbance regime [99]. Some studies have reported temporary shifts in microbial community diversity and composition following inoculation, but the long-term effects are less clear [100]. In some studies, microbial inoculants may positively interact with microorganisms of the same genus, enhancing the overall functioning of the soil ecosystem. This positive relationship highlights the potential of microbial inoculants to interact with existing microbial communities, leading to a more resilient soil ecosystem [20,101].

These techniques can also help track the population dynamics of specific bioinoculants. Papin et al. (2024) reported that inoculation density, rather than recurrent inoculation, had the strongest influence on the survival of a *P. fluorescens* B177 microbial inoculant in the soil [102]. However, recurrent inoculation had a limited impact on the diversity and composition of the resident soil bacterial community, while high density caused a small but significant decrease in bacterial richness and an increase in evenness, but no major shifts in community structure. These findings highlight the importance of optimizing inoculation density when applying bioinoculants to promote plant growth or exert their biocontrol mechanisms.

## 4. Challenges and Prospects

To date, research has provided strong evidence that PGPMs increase productivity through numerous mechanisms. The challenge for the scientific community and the industry is to develop tools and technologies that enable farmers to use these microorganisms effectively. Research efforts should focus on isolating new strains and developing bioformulations with a greater diversity of carriers and strains with different metabolic potentials, as well as diversifying the forms of application of the product to expand its use in all crops and different agricultural systems.

Recent studies highlight a positive relationship between plants and microbes in agricultural soils, underscoring the potential of leveraging these associations to isolate new PGPM strains. Domeignoz-Horta et al. (2024) demonstrated that increasing plant diversity enhanced positive microbial associations in the rhizosphere, which, in turn, improved microbial growth and carbon retention. These effects were attributed to greater resource complementarity and cross-feeding among microbes, facilitated by diverse root exudates. Such findings suggest that diverse cropping systems not only enhance carbon cycling dynamics but also promote the discovery of microbial communities with high CUE potential, offering new opportunities for the development of bioinoculants [103].

Scaling up PGPM production from laboratory to field conditions remains a challenge. Despite the recent progress in the field of production and formulation of microbial inoculants, additional studies are needed to further improve the upstream operational productivity, such as exploring alternative fermentation strategies, or different bioreactor configurations [34]. Implementing robust quality control and assurance protocols is vital to prevent errors and ensure the accuracy of results. This includes careful monitoring of critical parameters like microbial cell counts, viability, and formulation characteristics [5].

In terms of field efficiency, more research is needed to understand the complex interactions between PGPMs, plants, pathogens, and microbiota under field conditions, especially regarding the long-term effects of PGPMs on soil health and ecosystem functioning. In this sense, next-generation sequencing methods and multi-omics approaches should allow the identification, composition, and functions of the microbiome. A better understanding of these interactions will support more appropriate formulation, timing of application of biocontrol products, and integration with other agricultural practices.

The integration of PGPMs into organic farming systems exemplifies how sustainable agricultural practices can address environmental challenges while improving productivity. PGPMs contribute significantly to enhancing soil structure and increasing water-holding capacity, key factors in sustainable agriculture. By promoting soil porosity and aggregation through the secretion of extracellular polysaccharides and other bioactive compounds, PGPMs facilitate better water infiltration and retention, reducing runoff and soil erosion. Moreover, their ability to form symbiotic relationships with plants enhances nutrient uptake efficiency, preventing nitrate leaching into groundwater. These benefits align with the broader goals of integrating sustainable techniques such as organic farming with biocontrol and microbial inoculants, offering a comprehensive strategy to address environmental degradation while maintaining agricultural productivity [104].

The implementation of microbial inoculants into the market also needs to face the lack of awareness about the beneficial effects in terms of increased yield and productivity among crop producers. Even though the market potential of microbial inoculants was confirmed by recent analyses, which is estimated to be approximately USD 5.23 billion by 2029, with an annual growth rate of approximately 9.85% during the forecast period (2024–2029) for the biofertilizer market, and USD 11.41 billion by 2030 for the biopesticide market, with an annual growth rate of about 11.1% during the forecast period (2025–2030) [105,106], many growers perceive biocontrol products as more costly and less effective than traditional chemical products [107]. For this reason, it is crucial to enlarge the design of microbial inoculant research to cover the socioeconomic factors influencing the technology adoption, with the development of future multidisciplinary projects involving diverse actors throughout the value chain of agriculture.

The efficacy of microbial inoculants is another challenge due to their variability and dependence on environmental conditions, making it difficult for farmers to consistently achieve the desired benefits. In addition, extensive research is needed to study the effectiveness of inoculants in different crops while considering climate conditions and agricultural practice factors [5]. Moreover, integrating microbial solutions with other agricultural strategies in the context of climate change is essential [59,108]. To address variability, concrete strategies include selecting microbial strains with broad environmental adaptability, developing formulations that enhance microbial stability and activity under diverse conditions, as well as providing tailored recommendations for application timing, dosage, and crop compatibility [109,110]. These approaches, alongside continuous farmer education and robust field trials, can improve the consistency of results, ultimately increasing the approval and adoption of this technology by farmers.

On the other hand, the current regulatory requirements for PGPM products in agriculture are posing significant challenges to their successful commercialization and adoption by farmers. The regulatory framework could pose some bottlenecks for the sector development, including the fermentation, formulation phases, quality control, and efficiency validation [111]. As an example, in the case of biofertilizers, the European Union, in their most recent document, Regulation 2019/1009, currently limits the marketing to biofertilizers containing only four types of microorganisms (*Rhizobium* spp., *Azotobacter* spp., *Azospirillum* spp., and mycorrhizal fungi) [112]. Many countries also lack specific laws for biofertilizers and biopesticides, and their regulation is based on the laws for chemical fertilizers and pesticides [63]. Therefore, there is a need to update the regulatory framework to keep pace with the scientific advancements in PGPM. This will involve establishing clear guidelines for the development, testing, and registration of PGPM products to ensure their effectiveness and safety. Furthermore, public–private partnerships can play a key role in fostering innovation, funding large-scale trials, and building local expertise for production and application. Lastly, farmer awareness and education are critical to addressing the perception that microbial inoculants are less reliable than conventional agrochemicals. Training programs and extension services can disseminate best practices for inoculant use, emphasizing their long-term benefits in improving soil health and sustainability [113]. Integrating microbial inoculants into broader agricultural management strategies, such as conservation tillage or crop rotation, can also enhance their adoption and success.

## 5. Conclusions

The successful implementation of microbial inoculants will depend on the research of the advancements in production optimization, the understanding of the complex interactions between microorganisms and the environment, and the establishment of robust quality control measures. These efforts are crucial to fully exploit the potential of plant growth-promoting microorganisms in the development of efficient microbial inoculants tailored to diverse crops and agro-climatic conditions. Furthermore, strong and efficient collaboration between academia, industry, and policymakers is essential to create supportive regulatory frameworks and incentives that facilitate the commercialization and widespread adoption of this technology, thereby enhancing sustainable agricultural productivity and food security.

## Figures and Tables

**Table 1:** Medium and operation condition optimization for the cultivation of different microorganisms.

Microorganism.	Optimization Method	Culture Medium	Operation Conditions	Cultivation Method	Yield Max	References
*B. atrophaeus*	One-factor analysis, orthogonal experiments, and response surface methodology	Yeast extract, soy peptone, NaCl, NH_4_Cl, KH_2_PO_4_, Na_2_HPO_4,_ and MgSO_4_	28 °C, 3% inoculum volume, pH 7, 180 rpm, culture time 24 h	Submerged liquid fermentation.	1.4 × 10^10^ CFU/mL	[40]
*B. velezensis*	Plackett–Burman design and Box–Behnken design	Molasses as a carbon source, peptone as a nitrogen source, and MgSO_4_ as an inorganic salt	25.57 °C, 2% inoculum volume, pH 7.23, 160 rpm, culture time 95.90 h	Submerged liquid fermentation	3.39 × 10^10^ CFU/mL, and a bacteriostatic titer of 158.85 mm/m	[33]
*Trichoderma* sp.	Fractional factorial design and Box–Behnken method	Mixture of wheat bran and white rice	25 °C, 65% humidity, culture time 21 days	Solid-state fermentation	3 × 10^7^ spores/g dry substrate	[37]
*Cordyceps militaris*	One-factor analysis	Glucose and sucrose as carbon sources and yeast extract and casein hydrolysate as nitrogen sources	20 °C and a pH range of 4–6, in dark conditions, culture time 20 days	Submerged liquid fermentation	600–1000 mg/L	[41]
Exopolysaccharides of *Paenibacillus polymyxa*	One-factor-at-a-time approach and response surface methodology	Sucrose, yeast extract, K_2_HPO_4_, NH_2_NO_4_, KH_2_PO_4_, and MgSO_4_	30 °C, 10% inoculum volume, pH 7, 150 rpm, culture time 36 h	Submerged liquid fermentation	16.46 gL^−1^ (6.71-fold higher)	[42]
*Herbaspirillum seropedicae* and indole-3-acetic acid (IAA)	Response surface methodology	Glycerol and yeast extract	34–36 °C, 150 rpm and 4.0 L min^−1^ of airflow, 10% inoculum volume	Submerged liquid fermentation	3.4 gL^−1^ of biomass and 11.97 mgL^−1^ of IAA	[43]
*Purpureocillium lilacinum*	Response surface methodology and one-factor-at-a-time approach for central composite design	Wheat bran and yeast extract	67.98% moisture content, culture time 142.2 h	Solid-state fermentation	107.46 mg/g dry substrate (1.35-fold higher)	[44]

## Data Availability

The data about Biofertilizer and Biopesticide market can be found in Mordor Intelligence (2024) in the following links https://www.mordorintelligence.com/industry-reports/global-biofertilizers-market-industry/market-size/ accessed on 4 December 2024, https://www.mordorintelligence.com/industry-reports/global-biopesticides-market-industry/ accessed on 4 December 2024.
